# Supplementary material for: Causal Relationship between Aging and Anorexia Nervosa: A White-Matter-Microstructure-Mediated Mendelian Randomization Analysis
Source: Biomedicines. 2024 Aug 16;12(8):1874. doi: 10.3390/biomedicines12081874 (PMC11351342; doi:10.3390/biomedicines12081874)
Supplement: Supplementary file 1 [file biomedicines-12-01874-s001.zip › Supplementary Figure/Figure S8/Leaveoneout plot of MR analysis for TL to ubm-b-1526.pdf]

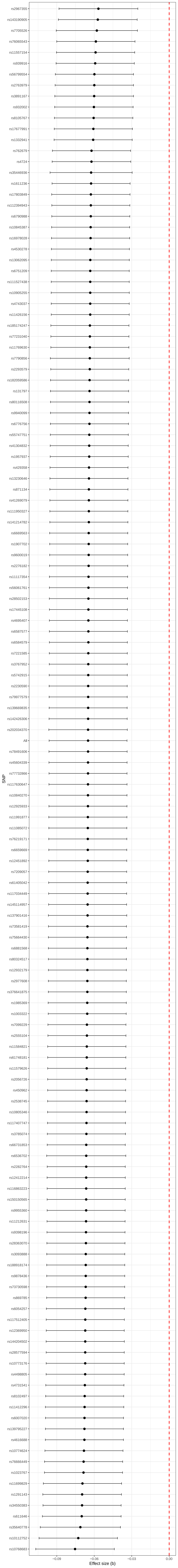
rs2967355
rs143190905
rs7705526
rs76065543
rs11557154
rs939916
rs56799554
rs2763979
rs3891167
rs932002
rs8105767
rs17677991
rs1332941
rs762679
rs4724
rs35446936
rs1611236
rs17803849
rs112394943
rs6790988
rs10845387
rs16978028
rs4530278
rs13062095
rs6751209
rs111527438
rs10905255
rs4743037
rs11426156
rs185174247
rs77231040
rs11769630
rs7790856
rs2293579
rs182059586
rs131797
rs80116508
rs9940099
rs6776756
rs55747751
rs41304832
rs1957937
rs429358
rs13230646
rs871134
rs41269079
rs111950327
rs141214782
rs6669563
rs1907702
rs9600019
rs2276182
rs11117354
rs56061761
rs28502153
rs17445108
rs4695407
rs6587577
rs6584579
rs7221585
rs3767952
rs5742915
rs2230590
rs79977579
rs139669835
rs142426306
rs202034370
All
rs78491606
rs45604339
rs77732866
rs117630647
rs10840270
rs12925933
rs11991877
rs11085072
rs76219171
rs6659669
rs12451892
rs7209057
rs61405042
rs117034449
rs145114957
rs137901416
rs73581419
rs75664430
rs6881568
rs80324517
rs12932179
rs2977608
rs376641875
rs1985369
rs1003322
rs7099229
rs2555104
rs11584821
rs61748181
rs11579626
rs2056726
rs450962
rs2538745
rs10805346
rs117407747
rs3785074
rs66731853
rs6536702
rs2282764
rs12412214
rs116863223
rs150150565
rs9955360
rs11212631
rs9398196
rs28363070
rs3093888
rs188918174
rs9878436
rs73730598
rs869785
rs6054257
rs117512405
rs12369950
rs144204502
rs28577594
rs10773176
rs4498805
rs4731541
rs8102497
rs11412296
rs6007020
rs139795227
rs4616688
rs10774624
rs76666449
rs1023767
rs11699829
rs1291143
rs34550383
rs611646
rs35640778
rs10112752
rs10768683
SNP
−0.09
−0.06
−0.03
0.00
Effect size (b)
